# Development, standardization and testing of a bacterial wound infection model based on ex vivo human skin

**DOI:** 10.1371/journal.pone.0186946

**Published:** 2017-11-15

**Authors:** Christoph Schaudinn, Christin Dittmann, Jana Jurisch, Michael Laue, Nazende Günday-Türeli, Ulrike Blume-Peytavi, Annika Vogt, Fiorenza Rancan

**Affiliations:** 1 Advanced Light and Electron Microscopy, Robert Koch Institute, Berlin, Germany; 2 Clinical Research Center for Hair and Skin Science, Charité –Universitätsmedizin Berlin, corporate member of Freie Universität Berlin, Humboldt-Universität zu Berlin, and Berlin Institute of Health, Charité, Berlin, Germany; 3 MJR PharmJet GmbH, Überherrn, Germany; LAAS-CNRS, FRANCE

## Abstract

Current research on wound infections is primarily conducted on animal models, which limits direct transferability of these studies to humans. Some of these limitations can be overcome by using–otherwise discarded—skin from cosmetic surgeries. Superficial wounds are induced in fresh ex vivo skin, followed by intradermal injection of *Pseudomonas aeruginosa* under the wound. Subsequently, the infected skin is incubated for 20 hours at 37°C and the CFU/wound are determined. Within 20 hours, the bacteria count increased from 10^7^ to 10^9^ bacteria per wound, while microscopy revealed a dense bacterial community in the collagen network of the upper wound layers as well as numerous bacteria scattered in the dermis. At the same time, IL-1alpha and IL-1beta amounts increased in all infected wounds, while—due to bacteria-induced cell lysis—the IL-6 and IL-8 concentrations rose only in the uninfected samples. High-dosage ciprofloxacin treatment resulted in a decisive decrease in bacteria, but consistently failed to eradicate all bacteria. The main benefits of the ex vivo wound model are the use of healthy human skin, a quantifiable bacterial infection, a measureable donor-dependent immune response and a good repeatability of the results. These properties turn the ex vivo wound model into a valuable tool to examine the mechanisms of host-pathogen interactions and to test antimicrobial agents.

## Introduction

Wound infections affect a large number of people (in 2009, 6.5 million patients in the USA) and their treatment eat up a considerable share of the annual health costs (in 2009, more than US$ 25 billion in the USA) [1]. The prerequisite to study wound infections and test new ways of treatments are meaningful model systems. Mouse, guinea pig, rat and rabbit models are among the most frequently used *in vivo* models for these studies [2–4]. Animal models are valuable tools to elucidate the mechanisms of wound infection in general. However, the anatomical and physiological differences in comparison to human skin (e.g. body hair, different immune response, thinner dermis and epidermis [5] limit their usability as models to realistically mimic chronic wound infections in men. Wound models that are based on pigskin have the charm that pig and human skin share a similar anatomy so that the obtained results are more likely to be also applicable to men [6]. Unfortunately, higher costs and more complex animal keeping requirements limit the practicability of pig-based wound models. Studies on humans are certainly the most accurate method to examine wound infections; however, ethical considerations will exclude a number of experiments right away (e.g. the use of untreated controls, sampling in triplicates, repeated biopsies, etc.)—and for the remaining studies, the number of volunteers with identical wound conditions will always be limited [6]. On the other hand, the number of cosmetic surgeries, in which excess fat is removed and discarded together with healthy skin, remains on a fairly high level (350,000 liposuction and abdominoplasty procedures in the USA in 2015) [7].

Ex vivo human skin has been used for years as model to study skin physiology and drug penetration [8, 9]. In previous studies, we employed ex vivo human skin to demonstrate its suitability for investigations of skin immune cells and their interactions with topically applied nanoparticles [10, 11]. This model was now adapted in order to reproduce some of the features of chronic wounds. Chronic wounds are very complex systems from both host and microbial point of view. It is hardly possible to reproduce all causal factors of chronic wounds. Nevertheless, it is now recognized that bacterial infections are one of the main driving factors for the development of a chronic wound [12]. Hence, in this work the main focus was given to bacterial infection, effects on immune response, and testing of antimicrobial treatments. Herein, we report on the development, standardization and testing of a bacterial wound infection model based on ex vivo human skin.

## Results and discussion

### Initial histologic examination of intact skin, induced wounds and infected wounds

**Fig 1:**
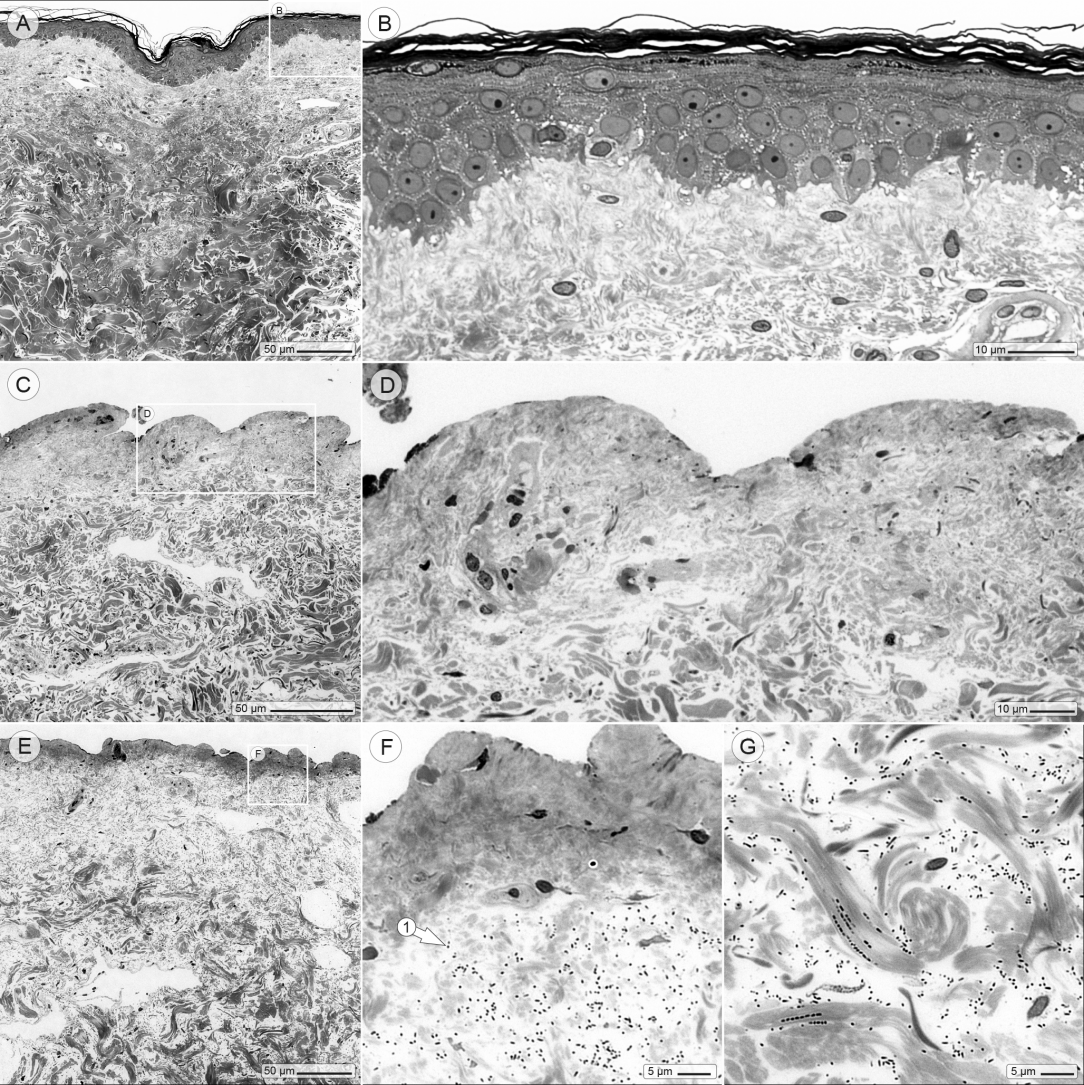
Initial histologic examination of intact skin, induced wounds and infected wounds. (A and B) Richardson stained section of intact human skin (abdomen), shortly after the cosmetic surgery. (C and D) Induced superficial wound: the epidermis was removed with a ball shaped milling cutter. (E-G) Wound, intradermally infected with *P*. *aeruginosa*. The bacteria (visible as black dots—arrow 1) are found scattered in the upper woundlayers (F) as well as in deeper wound regions (G).

In the first step, the histologic condition of the ex vivo skin was examined, followed by the distribution of the infecting bacteria. Whenever the ex vivo skin was left completely untreated after the surgical procedure, its histology revealed the well-known landscape of healthy skin (Fig 1A and 1B). Microscopy of the induced wounds confirmed that—as intended—exclusively the epidermis had been removed (Fig 1C and 1D). Shortly after the *P*. *aeruginosa* PAO1 strain was injected under the wound, the bacteria were either found loosely scattered throughout the dermis or associated with collagen bundles (Fig 1E–1G).

### Wound histology, twenty hours after the infection

**Fig 2:**
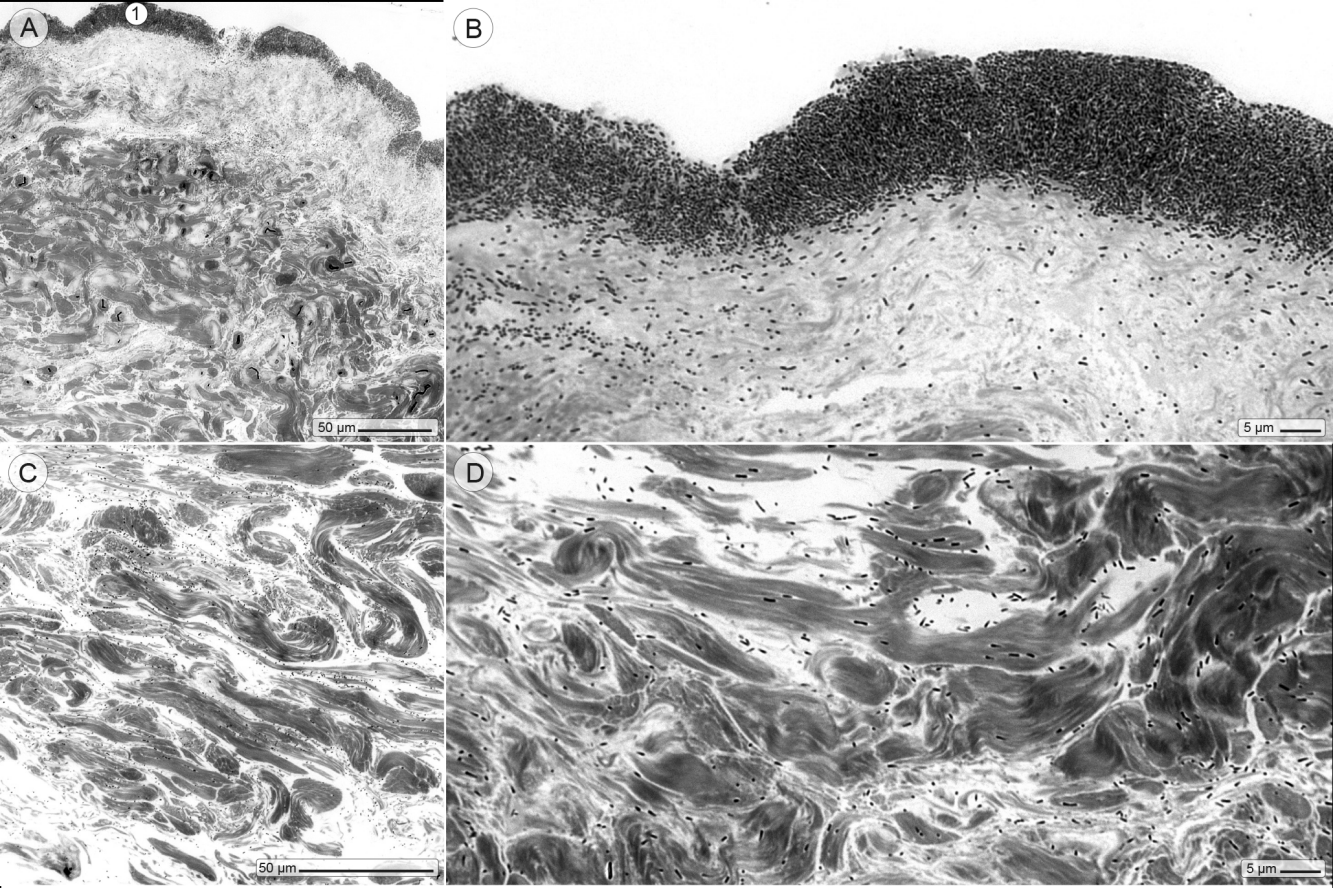
Wound histology in the light microscope, twenty hours after the infection. (A-B). The Richardson stained sections show a dense layer of bacteria that takes up the upper part of the wound (circle 1). (C and D) Lower skin layers—close to the subcutis—reveal scattered bacteria.

Twenty hours after the injection, the bacteria had multiplied, especially at the surface of the wound bed, where they were found associated with the collagen network (Fig 2A and 2B). Deeper skin layers also showed bacteria, albeit in much lower quantities (Fig 2C and 2D). *P*. *aeruginosa* is a preferentially aerobic growing bacterium [13]. The presence of oxygen at the top of the skin apparently enabled the bacteria there to achieve much higher growth rates than the bacteria in lower parts of the dermis. Although we focused on an infection with a single, strictly aerobe bacteria (*P*. *aeruginosa*), most chronic wound infections have a polymicrobial nature, in which strict and facultative anaerobe bacteria dominate [14].On the other hand, the *P*. *aeruginosa* PAO1 strain is a very well characterized bacteria so that our results could be easily compared to findings of other studies.

**Fig 3:**
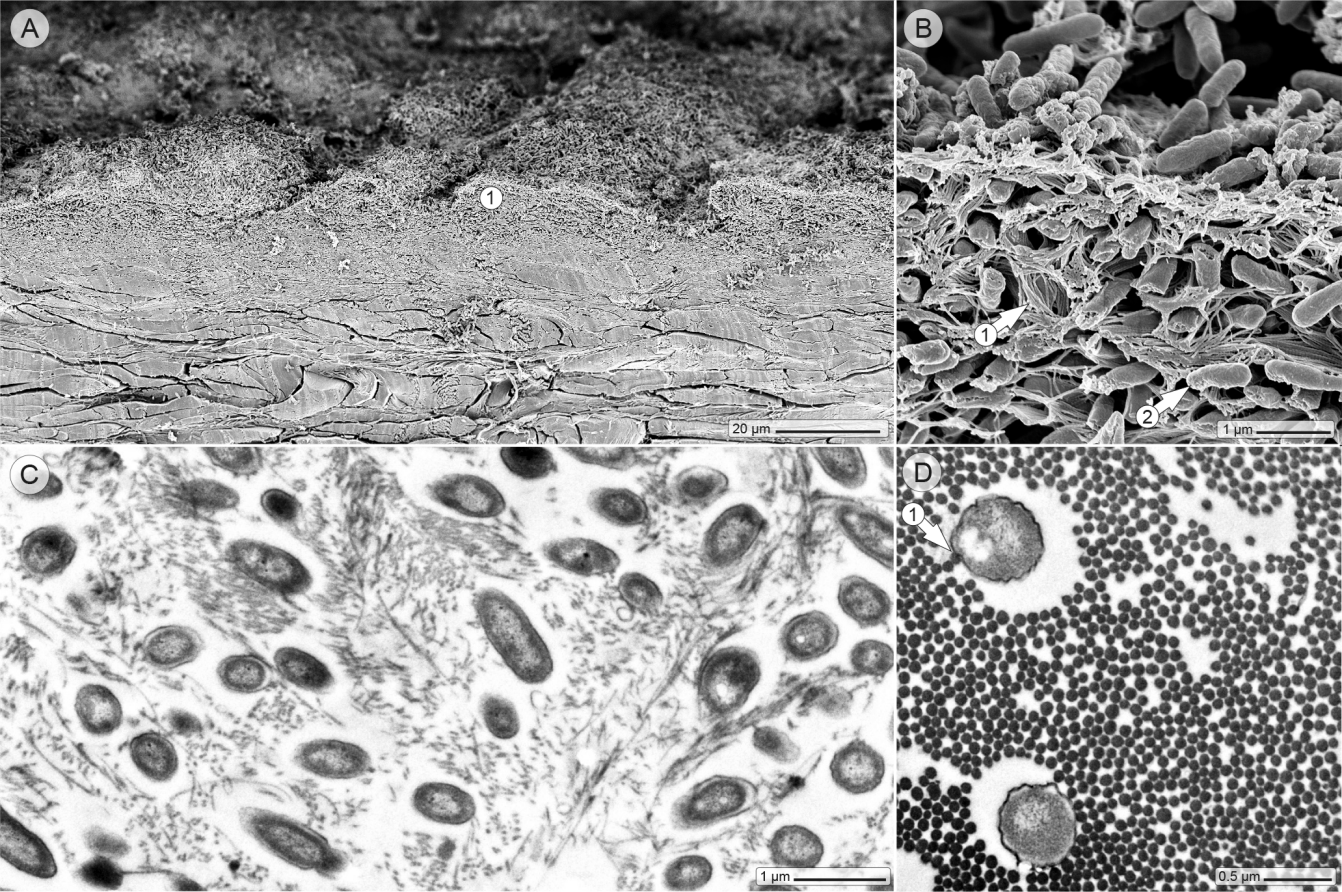
Wound histology in the electron microscope, twenty hours after the infection. (A) Scanning electron microscopic image of the skin wound profile: the wound surface is entirely covered with layers of bacteria (circle 1), (B) whose hill-like structures consist of collagen bundles (arrow 1) colonized by bacteria (arrow 2). (C and D) (Transmission electron microscopic images) Ultrathin sectioning reveals the organization of the bacteria within the collagen bundles and physical contact between bacteria and collagen fibers (arrow 1).

Scanning electron microscopy of vertically cut skin revealed numerous hill-like structures in the wound bed, which consisted of densely packed bacteria within a network of collagen fibers (Fig 3A and 3B). Transmission electron microscopy confirmed the high number of bacteria that were interwoven in collagen bundles and apparently physically attached to the collagen fibers (Fig 3C and 3D). Remarkably, the latter observation was also made in chronic wounds of affected patients [15]. Wounds can have either an acute or chronic status. However, a clear histologic separating line between both wound types could not be drawn. James et al. observed in chronic human wounds (persisting ≥ 30 days) “densely aggregated colonies of bacteria”, while bacteria in acute wounds tended to appear more as “individual cells or small microcolonies” [15]. In the ex vivo wound model, the appearance of individual bacteria deep in the dermis coincided with the hallmarks of an acute wound, while the appearance of the bacterial community on the wound surface bore more resemblance to characteristics of chronic wounds [15, 16].

### Imaging the spread of the infection

**Fig 4:**
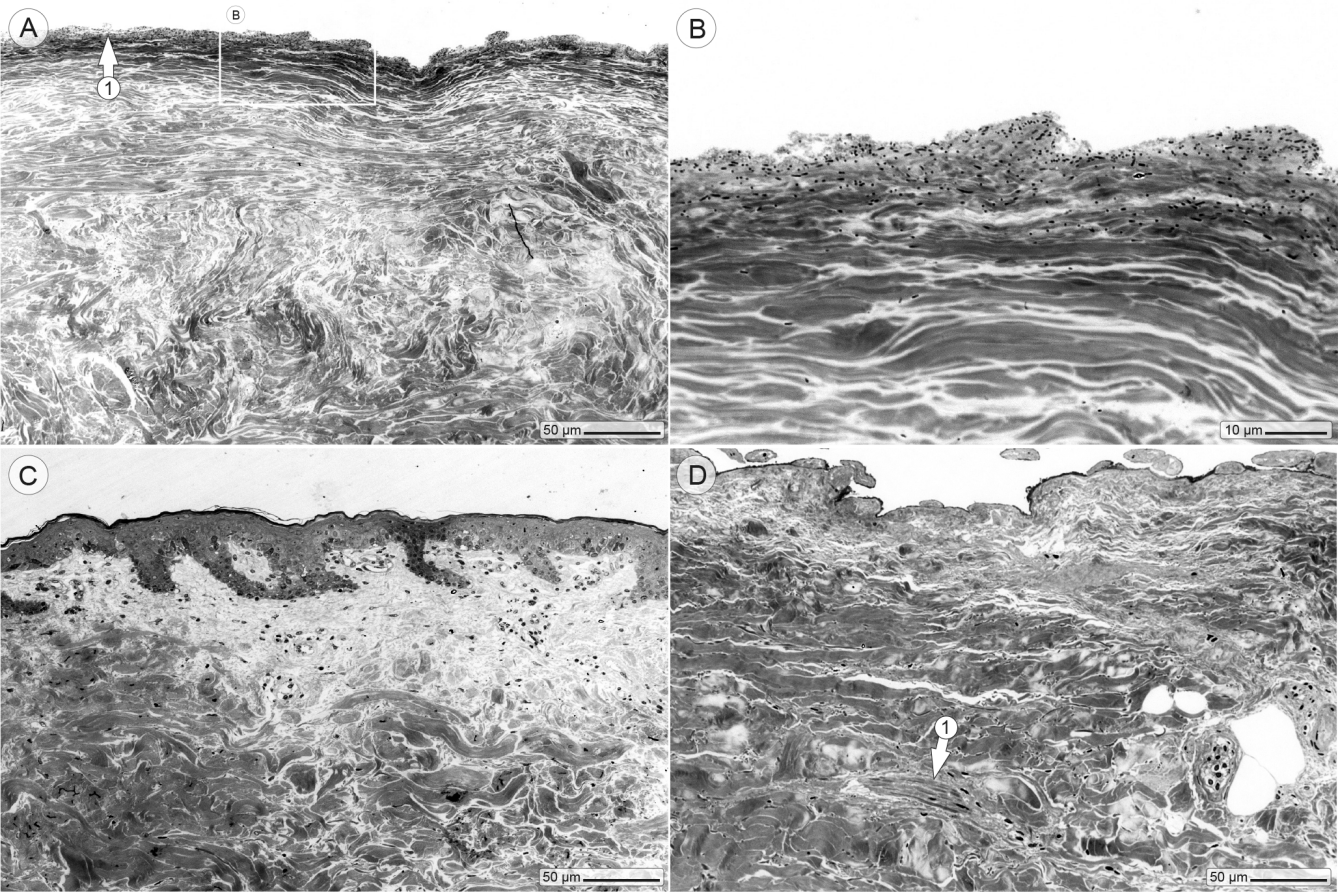
Imaging the spread of the infection. (A and B) The edge zone of the wound shows a thin bacterial layer on its surface (arrow 1). (C) Outside the wound, the skin morphology is intact and no obvious signs of an infection are visible. (D) All uninfected wound control samples still show common dermal cells after 20 hours (arrow 1).

The number of bacteria declined toward the edge of the wound, where merely a thin layer of bacteria was visible at the skin surface (Fig 4A and 4B). While absolutely no skin cells were observed in the center of the infected area, which suggests a massive cell lysis by the bacteria, some cells could be found in the dermis at the edge of the wound. Beyond the edge of the wound, completely intact skin morphology was regularly observed (Fig 4C), and a wide variety of skin cells could be found in all uninfected control wounds after 20 hours of incubation (Fig 4D).

Microscopic analysis of the bacterial distribution clearly demonstrated that the infection was limited to the confinements of the wound (which had typically a diameter of 5 mm). Consequently, an 8 mm biopsy of the wound provided a good safety margin and guaranteed high bacterial retrieval rates combined with a sufficient repeatability between different experimental runs.

### Bacteria quantification and antimicrobial treatment

**Fig 5:**
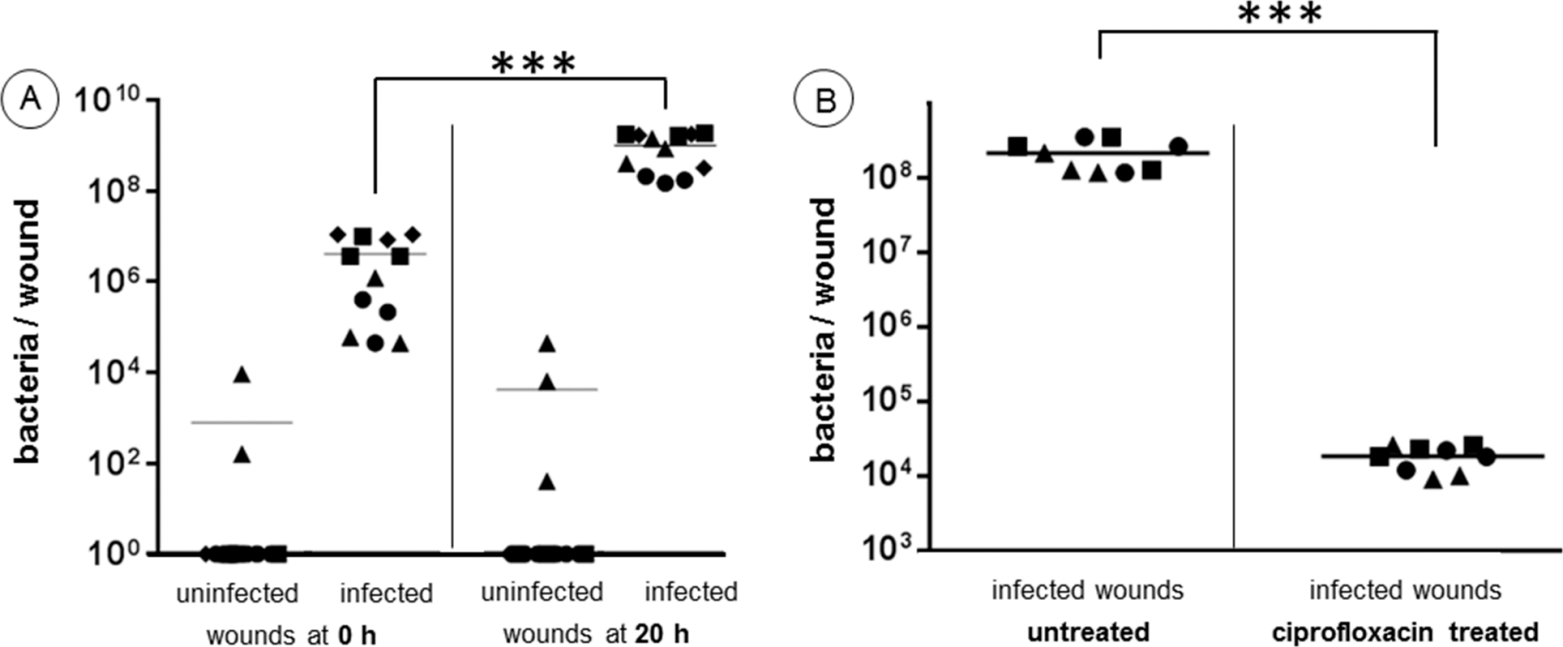
Bacteria quantification and antimicrobial treatment. (A) Bacteria counts of uninfected and infected wounds after 0 and 20 hours (●,▴, ■ and ◆ represent triplicates of donors 1–4). (B) Bacteria counts of infected wounds with and without topical ciprofloxacin treatment (10 μL, 375 μg mL^-1^, 20 hours,) (●,▴ and ■ represent triplicates of donors 5–7). *** p < 0.001.

Approximately 1·10^7^ bacteria were injected intradermally under a wound. While the mean number of bacteria that could be re-isolated at t_0 h_ for two donors ranged around 5·10^6^ bacteria/wound, the mean of the other two donors were closer to 5·10^5^ bacteria/wound (Fig 5A). The reason for these differences probably lies in the individual intensity of the immune response of each donor at this time, considering that otherwise the data points of a given donor are grouped quite closely.

Twenty hours later, the CFU count average had uniformly risen to approximately 1·10^9^ bacteria per wound. Pre-experiments, in which only 1·10^4^ bacteria were injected into the wounds, also resulted in CFU counts of 1·10^9^ bacteria per wound after 20 hours. The considerable increase in bacterial cell numbers after 20 hours hints toward an abundant presence of nutrients. Collagen represents the most frequent entity in the dermis. The here used *P*. *aeruginosa* PAO1 strain is known to produce a very powerful extracellular protease, which exhibited high collagenase activity in rats after surgical injury [17, 18]. Equipped with such a collagenase, the PAO1 bacteria can effectively convert collagen into nutrients.

Interestingly, only in one out of four donors, bacteria could be recovered from the uninfected controls (Fig 5A). These results suggest a thorough skin disinfection of each patient before surgery.

In order to explore the wound model’s potential as a test system for local antimicrobial treatment, the infected wounds were medicated with ciprofloxacin, which is frequently used as a topical treatment of *P*. *aeruginosa* caused ear infections [19]. The 20 hours ciprofloxacin treatment (375 μg mL^-1^) of infected ex vivo wounds resulted in a reduction from ~10^8^ to ~10^4^ bacteria per wound (Fig 5B). Wang et al. determined the minimal bactericidal concentration (MBC) for planktonic PAO1 bacteria toward ciprofloxacin with 0.125 μg mL^-1^, and the minimal biofilm eradication concentration (MBEC) for one day old biofilms (PAO1) with 16 μg mL^-1^ using the microtiter plate method [20]. As expected, PAO1 bacteria in their biofilm mode were significantly more tolerant (128-times) against the same antibiotic in comparison to their planktonic counterparts. Unexpectedly however, a slightly higher MBEC concentration of ciprofloxacin failed to kill all bacteria in the ex vivo wound model and let ~10^4^ bacteria survive. (A wound volume of ~125 mm^3^ was treated with10 μL of a 375 μg mL^-1^ solution, which corresponds to ~30 μg mL^-1^). This is even more surprising, considering that the overwhelming majority of bacteria at the wound bed surface were as directly exposed to the antibiotic as the biofilm bacteria at the bottom of the microtiter plate when the MBEC was determined.

The above observation strongly underlines the importance of experimental settings, which closely mirror the in vivo situation to be tested.

### Quantification of selected interleukins in infected and uninfected wounds

In order to investigate if a bacterial infection still can evoke an immune response in ex-vivo human skin, the concentrations of selected cytokines were determined. IL-6 and IL-8 were chosen because they are the most studied markers, whose expression is increased in inflammatory and infectious diseases. IL-1β is known to be secreted by several skin cell types, like macrophages and dendritic cells, when bacterial components bind to the Toll-like receptor 4. Finally, IL-1α was selected because it is related to tissue damage as well as to bacterial infections (IL-1 α was recently shown to induce dermal clustering of DCs with effector T cells during antigen presentation [21].

**Fig 6:**
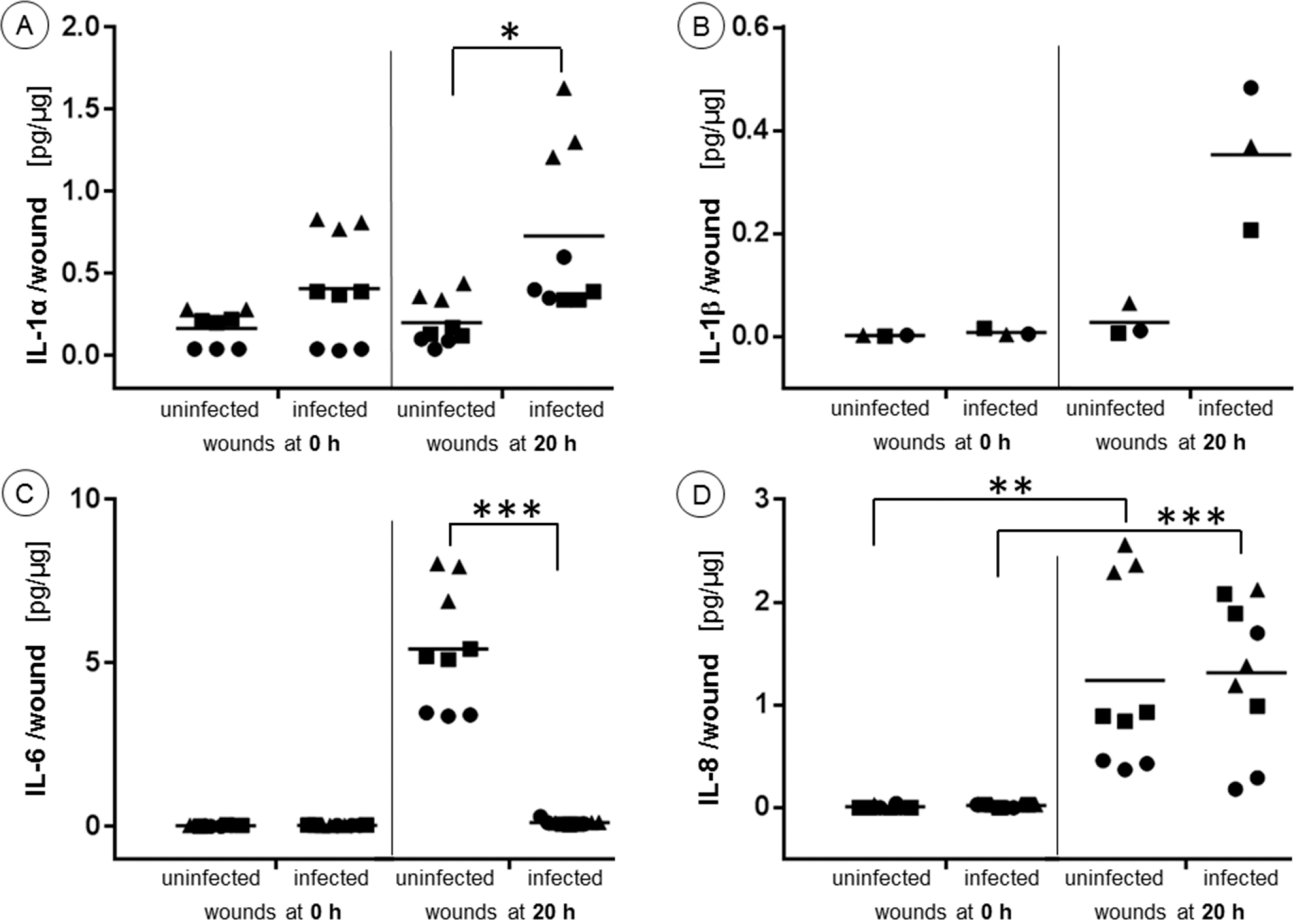
Quantification of selected interleukins in infected and uninfected wounds. (A) IL-1α, (B) IL-1β*, (C) IL-6, and (D) IL-8 concentrations of uninfected and infected wounds after 0 and 20 hours. (●,▴ and ■ represent triplicates of donors 1–3) (*Due to the limited volume of the testing material, triplicates had to be pooled). * p> 0.05, **p>0.01, *** p < 0.001.

As for IL-1α and IL-1β, their amounts were–in average—mostly increased after 20 hours of ongoing infection, while the uninfected controls resided generally at low levels. Only the IL-1α concentrations were already elevated in infected wounds from two of three donors at t_0 h_ (Fig 6A). Keratinocytes express permanently IL-1α [22]which allow for their immediate release in case of damage or infection–as the increased IL-1α levels at t_0 h_ confirm. After 20 hours, the graded IL-1α expression discriminates distinctly between infected and uninfected wounds, indicating a response of skin cells to the infection of the PAO1 strain.

Other than IL-1α the IL-1β release depends on the cleavage of pro-IL1β by caspase-1 upon the stimulation of keratinocytes, fibroblasts, macrophages and dendritic cells by bacteria [23]. Such a process requires more time, which is reflected in the still unchanged IL-1β levels at t_0 h_, while after 20 hours the IL-1β concentrations had eventually risen (Fig 6B).

In case of IL-6, no traceable amounts were found in any samples at t_0 h_, and neither in the infected samples after 20 hours. Only the uninfected samples at t_20 h_ showed a significant increase in IL-6 concentrations (Fig 6C). The IL-8 concentrations of all samples at t_0 h_ ranged below the detection limit, whereas the average IL-8 amounts at t_20 h_ had noticeably risen but showed no differences between infected and uninfected wounds (Fig 6D). Döge et al. found similar IL-6 and IL-8 levels in excised, uninfected human skin with mild barrier disruption [24], which suggests that the IL-6 and IL-8 amounts in uninfected ex vivo samples have to be credited solely to the damaged tissue.

As for the infected wounds after 20 hours, it was expected that the bacterial infection would have boosted the expression of IL-6 and IL-8, considering that the release of these cytokines by skin cells (fibroblasts and macrophages) is normally up-regulated during an infection [25]. On the other hand, it has been shown that the serine protease of PAO1 is able to degrade cytokines in acute infections, which results in a disruption of the host inflammatory response [26, 27]. Why specifically IL-6 was suppressed in case of the ex vivo wound model and not IL-8 remains to be explored.

## Conclusions

The here presented ex vivo wound model fills an interesting gap between the possibilities of classic animal models, attempts to create synthetic skin in vitro, and the very limited experiments that can be performed on human volunteers. The obvious benefits of this model are: a) the use of healthy human skin, b) a quantifiable immune response that can discriminate between individuals c) a standardized protocol, and d) relatively low-key ethical considerations. The most important potential of the ex vivo wound model lies in the possibility to study the dynamics of infections caused by bacteria—and possibly also of viruses, fungi, dermatophytes and parasites, combined with the option to test antimicrobial countermeasures for the treatment of chronic wound directly on human.

## Material and methods

### Skin sampling and processing

Appropriate institutional review board approval of the Ethics Committee at the Charité-Universitätsmedizin Berlin was obtained for this study (EA1/135/06, renewed in July 2015), and informed consent was acquired from each patient according to the declaration of the Helsinki guidelines. All skin samples were collected from healthy volunteers during cosmetic surgery, transported on 4°C cooling packs, and processed within 3–4 hours after surgery. The surface of the ex vivo skin (free of injuries or redness) was cleaned with double distilled water and trimmed to pieces of approx. 3x3x0.5 cm (length, width, height) with scissors in a way that a layer of 3–4 mm subcutaneous adipose tissue remained. Subsequently, the skin samples were mounted on aluminum foil wrapped Styrofoam pads using syringe needles. The epidermis was then removed with a rotating ball shaped milling cutter of 6 mm (No. 28725, Proxxon, Föhren, Germany) at 16,000 rpm, which was mounted on a dental micro motor handpiece (Marathon N7, TPC Advanced Technology, Inc. Diamond Bar, CA, USA) to induce a superficial wound of approximately 5x5 mm.

### Infecting the wound

The *P*. *aeruginosa* strain PAO1 (ATCC 15692), which was originally isolated from an infected human wound [28], was incubated for 20 hours in tryptic soy broth at 37°C on a shaker. A total volume of 5 μL (containing 1·10^7^ bacteria) was sucked into a 10 μL syringe (26 gauge) with tapered tip (#002000, SGE Analytical Science, Ringwood Victoria, Australia). The skin was pierced with the needle at the edge of the wound at a low angle (approx. 10 degree) and then pushed toward the wound center, where the bacteria were ejected.

Severe wound infections often show that bacteria have invaded deeper skin layers [2, 15]. In order to get closer to these kinds of wound infections, we decided to inject the bacteria intradermally, rather than deposit them on top of the wound.

As a control, and in order to determine the number of skin-innate bacteria, 5 μL sterile saline (0.9% NaCl) was injected under the wound as described above.

### Determination of CFU counts

Each tested setting was done in triplicates with a total of at least three runs. The skin was either immediately processed (t_0 h_) or incubated in a moisture chamber (Emsa clip and close storage container, Emsa, GmbH, Emsdetten, Germany) for 20 hours at 37°C (t_20 h_). Using an 8 mm biopsy punch, the 5 mm wound, including some surrounding intact skin, was excised and placed in a 1.5 mL microcentrifuge tube containing 0.2 mL saline. The skin-pieces were then homogenized for 3 min with a sterile steel pistil at 150 rpm mounted on a digital overhead stirrer (DSL, VELP Scientifica Srl, Usmate, MB, Italy). Afterwards, all samples were sonicated in an ultrasonic bath (BactoSonic®, Bandelin, Berlin, Germany) at 40 kHz for 10 min using 200 W_eff_ to detach the bacteria from the tissue and individualize the bacteria. 100 μL of each sample was transferred to the respective wells of the ‘A’ rows of 96 well microplates, while the rest of the samples in the microcentrifuge tubes were stored at -80°C for ELISA analysis. The samples in the 96 well microplates were then diluted from row ‘A’ down to row ‘H’ in 1:10 steps (20 μL sample + 180 μL saline) by using a multichannel pipette. 5 μL of each well was spot deposited with a multichannel pipette on square tryptic soy agar plates. The plates were incubated overnight at 37°C and spotting areas with 5 to 50 CFU counted [29].

### Ciprofloxacin treatment

The infection of skin samples was done as described above. Ciprofloxacin (Sigma-Aldrich Chemie GmbH, Munich, Germany) was solved in double distilled water to a final concentration of 375 μg mL^-1^ (This was the highest possible concentration at pH 7.0). Twenty hours after the injection with *P*. *aeruginosa*, 10 μL of the ciprofloxacin solution was placed on top of each infected wound, and subsequently incubated in a moisture chamber for another 20 hours at 37°C. Afterwards, CFU was determined as described above.

### Preparation for transmission electron microscopy

Three skin specimens of each setting were processed for transmission electron microscopy.

Two millimeter biopsies were taken (a) from the center of the wound (at t_0 h_ and t_20 h_), (b) directly at the edge of the wound (at t_20 h_), and (c) in close proximity to the wound (at t_20 h_, area with intact epidermis) and fixated in 4% formaldehyde (in 50 mM HEPES) for 24 hours at room temperature. The skin pieces were washed (50 mM HEPES) and incubated for 10 min in 50% ethanol at room temperature. All skin samples were then dehydrated on ice in 70, 100, 100% ethanol (10 min each step), infiltrated with a LR White-ethanol solution (equal volumes, 10 min.), and incubated again with pure LR White (2x15 min). Subsequently, the skin pieces were transferred to polyallomer centrifuge tubes (5x 20 mm, No.342630, Beckman Coulter, Inc. Brea, CA, USA), containing LR White with accelerator (5 μL mL^-1^ monomer). The centrifugation tubes were capped with gelatin capsule and polymerized for 1 hour on ice, and then incubated at 60°C overnight. Sections of ~75 nm were made for TEM imaging at the ultramicrotome (EM UC7, Leica, Wetzlar, Germany), stained with a solution of 0.9% uranyl acetate and 0.1% methyl cellulose (w/v) for 10 min, and imaged in the transmission electron microscope (Tecnai 12 Spirit; FEI, Eindhoven, Netherlands).

### Preparation for light microscopy

Three skin specimens of each setting were processed for transmission electron microscopy.

Five hundred nanometer sections from the LR White embedded samples were made for light microscopy at the ultramicrotome, mounted on poly-L-lysine slides and placed for 10 min on an 80°C thermoplate. Staining was done for 4 min with Richardson’s stain, then washed with ddH_2_O, and imaged in the microscope (Axiophot, Carl Zeiss Microscopy GmbH, Germany).

### Preparation for scanning electron microscopy

Three skin specimens of the infected wound at t20h were processed for scanning electron microscopy. Skin preparation and infection was done as describe above. After the skin was incubated for 20 hours at 37°C in a moisture chamber, 8 mm biopsies were taken and fixed in a solution of 4% formaldehyde and 0.5% glutaraldehyde (in 50 mM HEPES) for 48 hours at room temperature. One of the sample duplicates was sliced right through the wound area with a scalpel in order to reveal the skin profile. All skin samples were afterwards washed in 50 mM HEPES, dehydrated in 30, 50, 70, 90, 95, 100, 100% ethanol, critical point dried, mounted on aluminum stubs, sputter coated with a 12 nm layer of gold-palladium and finally examined in the SEM (ZEISS 1530 Gemini, Carl Zeiss Microscopy GmbH, Germany) operating at 3 kV using the in-lens electron detector.

### Image processing

Images have been cropped, adjusted for optimal brightness and contrast (applied to the whole image) using Photoshop Lightroom® (Adobe Systems, San Jose, CA, USA).

### Protein extraction and enzyme-linked immunosorbent assay (ELISA)

Each tested setting was done in triplicates with a total of three runs.

All samples that were designated for protein extraction were stored at -80°C after the sonication step until further processing. After thawing, 200 μL extraction buffer (100 mM Tris-HCl, 150 mM NaCl, 1 mM EDTA, 1g Triton X-100) was added and samples were vortexed for 8 seconds. The samples were then incubated under constant shaking (600 rpm) for 30 min at room temperature and centrifuged for 10 min at 450 x g. The supernatant was aliquoted and used for cytokine measurements. Human IL-1α, IL-1β, IL-6, and IL-8 were examined using ELISA kits (CytoSetTM, Invitrogen Corp., Carlsbad, CA, USA). The values were normalized to total soluble protein content determined by the Pierce 660 nm protein assay (Thermo Scientific Inc., Rockford, IL, USA). All kits were used according to the manufacturer’s recommendations. Absorbance values were measured with a plate reader (EnSpire® Multimode Perkin Elmer, Akron, OH, USA).

### Statistical analysis

In total, skin from 12 donors was used. Each experiment was repeated at least three times using skin from different donors. Each control sample was done in triplicate. The Excel software (Microsoft, Redmond, WA, USA) was applied for statistical analysis. One way ANOVA and two-tailed unpaired student-T tests were used. For data of Fig 5 there was a statistical significant difference between the four groups in A and the two groups in B (F = 2.54 and 4.49, respectively). For data in Fig 6, F values of 4.14 were found for IL-1α, IL6, and IL-8; p values were reported in figures as follows: p> 0.05, **p>0.01, *** p < 0.001. Only p ≤ 0.05 was regarded as a significant difference.
